# Analysis of *in situ* Transcriptomes Reveals Divergent Adaptive Response to Hyper- and Hypo-Salinity in the Hong Kong Oyster, *Crassostrea hongkongensis*

**DOI:** 10.3389/fphys.2018.01491

**Published:** 2018-10-26

**Authors:** Shu Xiao, Nai-Kei Wong, Jun Li, Yue Lin, Yuehuan Zhang, Haitao Ma, Riguan Mo, Yang Zhang, Ziniu Yu

**Affiliations:** ^1^CAS Key Laboratory of Tropical Marine Bio-Resources and Ecology, Guangdong Provincial Key Laboratory of Applied Marine Biology, South China Sea Institute of Oceanology, Chinese Academy of Sciences, Guangzhou, China; ^2^State Key Discipline of Infection Diseases, Shenzhen Third People’s Hospital, Shenzhen, China

**Keywords:** oysters, *Crassostrea hongkongensis*, adaptive plasticity, salinity, osmoregulation, transcriptomics

## Abstract

*Crassostrea hongkongensis*, a commercially valuable aquaculture species dwelling in estuaries along the coast of the South China Sea, is remarkable for its eurysalinity traits that enable its successful colonization of diverse osmotic niches ranging from near freshwater to seawater. In order to elucidate how this oyster copes with coastal waters with immense salinity differences, we performed *in situ* transcriptomic analysis (RNA-seq) to characterize the global expression patterns of oysters distributed across naturally formed salinity gradients in Zhenhai Bay along the northern coast of the South China Sea. Principal component analysis reveals distinct expression profiles of oysters living in the extreme conditions of hypo-salinity and hyper-salinity. Compared with the situation of optimal salinity for oyster growth, hypo-salinity mainly regulated expression of genes involved in FoxO and oxytocin signaling, tight junction and several immune pathways, while hyper-salinity altered gene expression implicated in amino acid metabolism, AMPK and PI3K-AKt signaling pathways, demonstrating the complexity and plasticity of transcriptomic expression underpinning oyster eurysalinity. Furthermore, the expression patterns of several genes correlated with salinity gradients reveals the fine-tuned coordination of molecular networks necessary for adaptive homeostasis in *C. hongkongensis*. In conclusion, a striking capacity and distinct patterns of transcriptomic expression contribute to eurysalinity adaptation in *C. hongkongensis*, which provides new mechanistic insights into the adaptive plasticity and resilience of marine mollusks.

## Introduction

Estuaries are dynamic and intricate ecosystems characterized by rapid periodic fluctuations in physical parameters such as salinity, oxygen levels and temperature. To cope with these challenging environmental oscillations, local marine organisms have evolved various adaptive strategies to enable survival and physiological plasticity. The Hong Kong oyster, *Crassostrea hongkongensis*, is an endemic and commercially valuable aquaculture species that thrives along the northern coast of South China Sea (Lam and Morton, 2004). As benthic and sessile filter-feeders, the Hong Kong oyster has acquired a powerful capacity for adapting to the inherently dynamic estuarial habitats (Liu et al., 2013). Notably, this oyster exhibits remarkable eurysalinity traits, which allow it to survive in extreme conditions across salinity gradients from seawater down to near freshwater, making it one of the predominant estuarial inhabitants in the local region. The Hong Kong oyster typically thrives within a salinity range of 5–30 ppt and the optimal salinity levels for it are approximately 10–25 ppt. It therefore exhibits adaptive plasticity superior to that of common species such as *Crassostrea giga*, *Crassostrea angulata* and *Crassostrea sikamea* (Lam and Morton, 2004; Liu et al., 2013). Previous findings have shown that substantial genetic variation could arise across the various oyster populations cultivated in different salinity niches (Li et al., 2013; Ma et al., 2016). Therefore, the Hong Kong oyster serves as an excellent model for exploiting the molecular mechanisms underlying functional plasticity in response to extreme osmotic stresses in bivalves.

Recently, several studies have employed transcriptomic analysis to investigate the molecular basis of oyster osmotic regulation, though their scope has been limited to experimental manipulations within the laboratory (Meng et al., 2013; Zhao et al., 2014; Yan et al., 2017). Currently, there is a paucity of understanding about the regulatory mechanisms of oyster salinity acclimation *in situ*. In this present study, we have carefully designed field experiments to sample oysters from different osmotic niches which encompass a continuum from freshwater to seawater, with the explicit aim of capturing snapshots of active transcripts under *in situ* conditions. Zhenhai Bay in Guangdong province, China, was chosen as a representative estuary area of the northern South China Sea for field study, where salinity gradients are naturally formed along the coast and punctuated with seasonal changes. Hong Kong oyster’s success to thrive in local intertidal niches has been attributed to a strong and vertically inherited capacity for osmotic regulation, eventually leading to the emergence of endemic populations of this species. This coastal area thus provides us an ideal sampling location to study salinity adaption of oysters. This study represents a systematic attempt to understand the molecular underpinnings of oyster adaptive response to extreme conditions of salinity *in situ*. Analysis of *in situ* transcriptomes was used accordingly to characterize the genomic response to extreme osmotic conditions, with the hope of gaining insights into the crucial molecular determinants or pathways that contribute to govern the physiological plasticity in the Hong Kong oyster.

## Materials and Methods

### Ethics Statement

*Crassostrea hongkongensis* is neither an endangered nor protected species. Oyster handling was conducted in compliance with the guidelines and regulations established by the ethics committee of the CAS South China Sea Institute of Oceanology and the local government.

### *In situ* Sample Collection

*In situ* oyster sampling was conducted in Zhenhai Bay, situated between the Wencun and Beidou towns in Taishan municipality, Guangdong province, China. Its specific geographical coordinates are 21.44–21.95° north latitude, and 112.24–112.65° east longitude. The bay has in excess of 80 km of shoreline with an array of substrates including clay, mud, sand, and concrete from urbanized areas. It is the only estuary of the local Nafu River.

The oysters used in this study were collected at November 12, 2017 from six locations within the salinity gradient map of the local coast. The overall sample collection comprised of 90 wild 1-year old oysters (15 oysters per site). The shell height (maximal hinge-lip distance) of the oyster sampled ranged from 6 to 8 cm. The gills of the oysters were processed for total RNA isolation *in situ* within 30 min after their harvest on site.

### RNA Isolation From Gills

Total RNA was isolated from the gills of oysters collected from different locations by using TRIzol Reagent (Invitrogen, United States) following the manufacturer’s instructions. The isolated RNA was incubated with RNase free DNase (Invitrogen, United States) to eliminate the potential genomic RNA contamination. The purity and concentration of RNA samples were determined by Nanodrop 2000 (Thermo Fisher Scientific, United States). RNA integrity was verified by using an Agilent 2100 BioAnalyzer (Agilent, United States) with an RIN (RNA integrity number) setting >8.5. The same RNA samples from five oyster at each site was pooling as one biological replication, and each site contains three biological replications.

### Library Construction and RNA-seq

The cDNA library for Illumina sequencing was prepared by using a Truseq^TM^ RNA sample prep kit (Illumina). Briefly, mRNA was first enriched by using poly-T oligo-attached magnetic beads (Illumina), which was subsequently broken down into fragments (200–700 nt). The first and second strands of cDNA were synthesized by SuperScript II reverse transcriptase and DNA polymerase I, respectively. After that, the libraries were end-repaired and ligated with adaptors, and amplified with Phusion DNA polymerase. The quality of cDNA library was validated by TBS380 (PicoGreen) and the samples were then sequenced on a BGISEQ-500 sequencing platform (BGI, China).

### Real-Time qPCR Validation

One microgram of total RNA was used in reverse transcription (RT) experiments with PrimeScript^TM^ RT Reagent Kit (TaKaRa, Japan). All primers used in this studies were listed in Supplementary Table S4. The real-time qPCR was performed at the platform of LightCycler 480 (Roche) with 20 μL reaction system including 10 μL of 2× Master Mix (Roche), 0.4 μL of each of primers (10 mM), 5 μL of 1:50 diluted cDNA, and PCR-grade water for the rest. The dissociation curve analysis was performed to confirm specificity of amplicons. Each sample was carried out in triplicates, and relative expression levels of target genes was calculated using methods of 2^−ΔΔCt^ through normalized with reference gene GAPDH. Results were expressed as mean of log2 (fold-changes) to compare with the result obtained from RNA-seq.

### Bioinformatics Pipeline

#### Data Filtering

Clean reads were obtained by filtering raw data with the software SOAPnuke, which include removal of adaptors, unknown base reads (unknown bases were more than 10%) and low quality reads (cases were the percentage of base whose quality is less than 15% or greater than 50% in a read). Clean reads were stored in the FASTQ format and used for quantitative analysis.

#### Gene Expression Analysis

Clean reads were mapped onto known *C. hongkongensis* transcriptome datasets (Tong et al., 2015) by using Bowtie2 (Langmead and Salzberg, 2012), and the resultant matched reads were calculated and normalized to RPKM by using the RESM software (Li and Dewey, 2011). Pearson correlation coefficients between whole samples were calculated by using R cor, and PCA (principal components analysis) was performed by using princomp. Statistical significance of differentially expressed genes (DEGs) was analyzed via DEseq2 methods based on negative binomial distribution with the following threshold settings: fold change ≥2.00 and adjusted *p*-value ≤ 0.05 (Love et al., 2014).

#### Gene Ontology Analysis

Differentially expressed gene were subjected to analyses of Gene Ontology (GO) and KEGG Orthology (KO), which were further classified according to official classification. GO functional enrichment was performed by using phyper of R and the *p*-value was calculated in hypergeometric test with the following formula:

$$P=1-\sum_{i=0}^{m-1}\frac{\left(\frac{M}{i}\right)\left(\frac{N-M}{n-i}\right)}{\left(\frac{N}{n}\right)}$$

Then, false discovery rate (FDR) was calculated for each *p*-value, and we defined FDR <0.01 as significantly enriched (Ye et al., 2006; Kanehisa et al., 2008).

## Results

### Broad-Spectrum Tolerance of *C. hongkongensis* for Salinity Extremities

**FIGURE 1:**
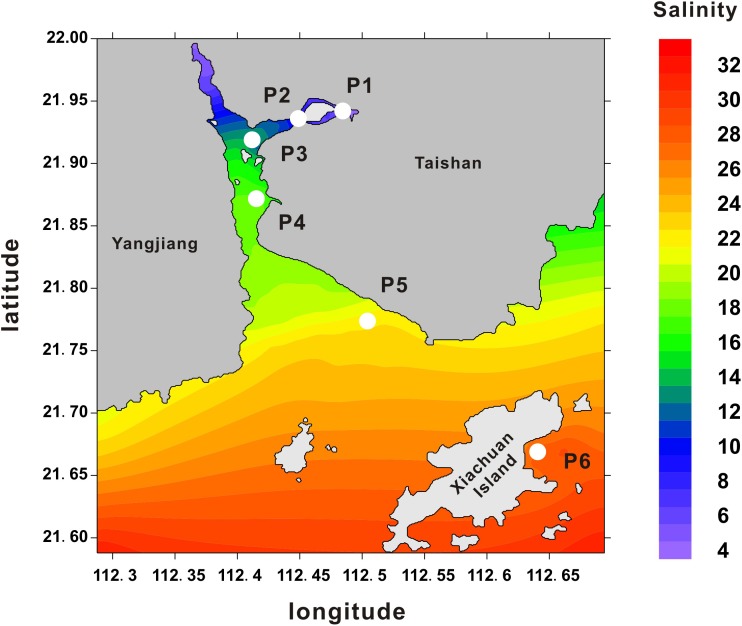
Oyster sampling locations across salinity gradients in Zhenhai Bay. Heatmap shows the average salinity (ppt) measured in different sampling areas between Shenjing town and Xiachuan Island. The salinity data were collected from the local Taishan Marine and Fisheries Agency. Numerals in the *y*- and *x*-axis indicate the coordinates in latitude and longitude, respectively.

*Crassostrea hongkongensis* is one of the euryhaline species that can survive under extreme conditions of salinity ranging from 2 to 30 ppt, and preferentially live in estuarial habitats. Zhenhai Bay features naturally formed salinity gradients comprising near freshwater and seawater. The geographic coordinate and annual mean salinity were presented Figure 1, including P1 (21.94N, 112.48E, 5.0 ± 3.9 ppt), P2 (21.93N, 112.45E, 8.4 ± 4.1 ppt), P3 (21.92N, 112.42E, 13.7 ± 5.2 ppt); P4 (21.87N, 112.43E, 19.2 ± 5.1 ppt); P5 (21.77N, 112.52E, 23.1 ± 3.9 ppt); P6 (21.67N, 112.64E, 28.1 ± 4.2 ppt). Here, *C. hongkongensis* occupied niches of diverse osmotic conditions across this bay. To characterize the molecular mechanisms underlying salinity adaption of the Hong Kong oyster, six distinct oyster populations (P1–P6) were collected across these salinity gradients. The realtime salinity were P1, 5.5 ppt; P2, 8.8 ppt; P3, 13.5 ppt; P4, 18.3 ppt; P5, 22.4 ppt; P6, 28.5 ppt, which was consistent with annual mean salinity by and large.

### Expression Patterns of Whole Transcriptomes

**FIGURE 2:**
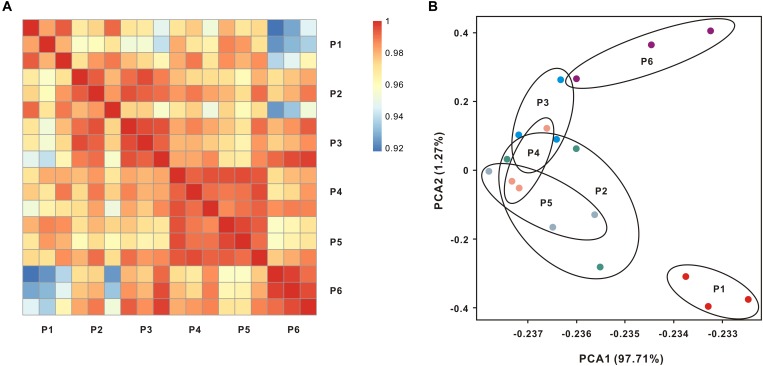
Global transcript expression profiles of oysters living across salinity gradients. **(A)** Expression patterns of whole transcriptomes were analyzed by means of RNA-seq quantification. The heatmap shows the square of correlation between oysters collected from different locations. Each location contains three independent biological replicates. **(B)** Principal components analysis (PCA) using the normalized whole transcriptome expression.

In our experimental design, 18 libraries were constructed from six populations. Each population contained three biological replicates. After sequencing and low quality filtration, a total of 352.65 million clean reads were obtained with an average of 24 million reads for individual samples (Supplementary Table S1), and the raw data were deposited into database with accession number (SRR7777763–SRR7777768). The percentages of total mapped reads and unique mapped reads were 76.79–83.15% and 73.59–80.64%, respectively (Supplementary Table S1). The Pearson correlation analysis of global expression profiles reveals that was observed a higher correlation within each population than that between different populations (Figure 2A and Supplementary Figure S1). PCA further suggests that the expression profiles of oysters collected from locations P2–P5 (salinity from 8.8 to 22.4 ppt) overlap with each other (Figure 2B).

### Pathway Enrichments in Hypo-Salinity and Hyper-Salinity Adaptation

**FIGURE 3:**
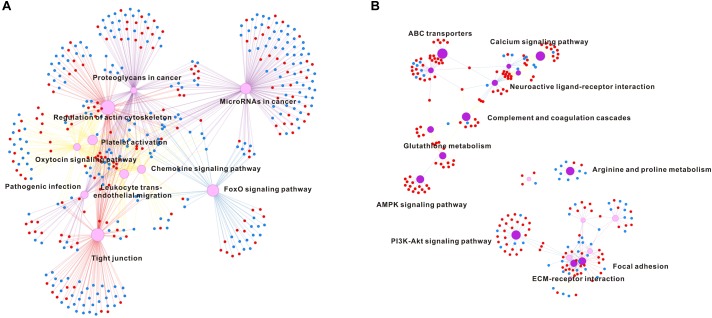
Pathway enrichment analysis in hypo-salinity **(A)** and hyper-salinity **(B)** adaptation. The top 10 enriched pathways are shown in the network. The up-regulated and down-regulated genes are indicated in red dot and blue dot, respectively.

To analyze which genes or pathways were involved in response to hypo-salinity and hyper-salinity adaptation, the DEGs obtained via the DEseq2 methods with samples at P3 as a control. Accordingly, hypo-salinity (P1 vs P3) generated 3,731 of up-regulated DEGs and 3,042 of down-regulated DEGs, whereas hyper-salinity (P6 vs P3) resulted in 641 of up-regulated DEGs and 563 of down-regulated DEGs (Supplementary Figures S2, S3). Furthermore, we classified these DEGs into different signaling pathways using KEGG (Supplementary Tables S2, S3). The top 10 enriched pathways in response to salinity extremities are listed in the Figure 3 to illustrate that hypo-salinity regulates many signaling pathways or processes, which include the FoxO signaling pathway, oxytocin signaling pathway, regulation of actin cytoskeleton, and tight junction. Concomitantly, several immune pathways are also influenced by hypo-salinity, including chemokine signaling pathway, pathogenic infection, platelet activation, etc. (Figure 3A). In contrast, oysters under hyper-salinity seem to follow a different pattern of regulation, which features pathways involved in arginine and proline metabolism, riboflavin metabolism, PI3K-Akt signaling, protein digestion and absorption, ECM receptor interaction, focal adhesion, neuroactive ligand receptor interaction and so on (Figure 3B). There are almost no identical categories between hypo- and hyper-salinity, implying that different regulatory mechanisms may be at work for salinity adaptation under distinct conditions.

### Osmotic Marker Genes

**FIGURE 4:**
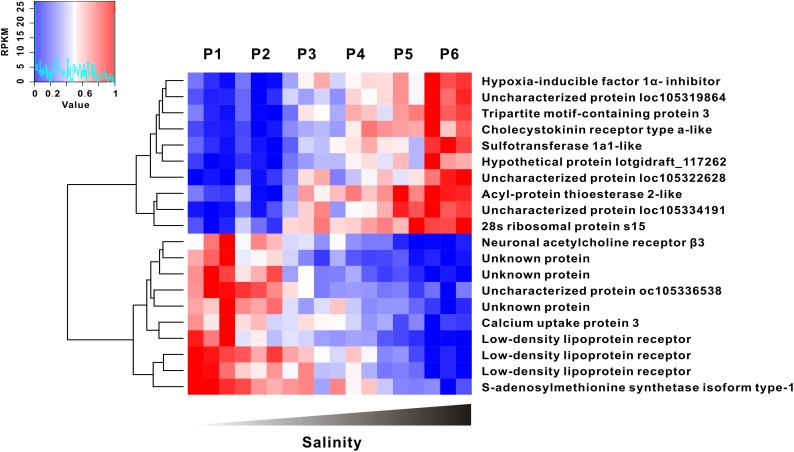
Gene expression was highly correlated with salinity gradient. The top 10 of salinity positive and negative correlation genes were clustered (|*R*^2^| > 0.89, *p* < 0.01).

To precisely identify which genes are directly correlated with the oyster response to extreme conditions along the salinity gradients (that is, osmatic marker genes), we performed a whole transcriptomic correlation analysis between gene expression and salinity gradient. The heatmap shows the top 10 genes positively or negatively correlated with salinity extremities (Figure 4). Notably, the expression level of hypoxia inducible factor 1 α inhibitor, cholecystokinin receptor type α like and sulfotransferase 1α1-like gene significantly increased with salinity elevation, whereas the expression level of low density lipoprotein (LDL) receptor, calcium uptake protein 3 and neuronal acetylcholine receptor (AChR) β3 dramatically declined as salinity increased. All of these genes showed a high correlation with the salinity gradient (*p* < 0.01), strongly suggestive of functional importance as marker genes in osmotic adaptation.

### RNA-Seq Validation by Real-Time qPCR

To validate the reliability of RNA-seq, 10 genes were selected to compare the expressional consistency between RNA-seq and Realtime PCR, including half of up-regulated DEGs and half of down-regulated DEGs. The scatter plot showed that the gene expression level are highly consistent (*R*^2^ = 0.912, Pearson’s correlation analysis, Supplementary Figure S4). Meanwhile, the references genes GAPDH wasn’t affected by salinity fluctuation.

## Discussion

In estuaries, osmoregulation is an essential and highly complex process through which oysters sustain their physiological activities despite dramatic variation in salinity and diverse osmotic niches. Previous studies have attempted to investigate the osmotic adaptive response based on experimental models mimicking salinity stress in laboratory environments (Meng et al., 2013; Zhao et al., 2014; Yan et al., 2017). However, there is a dearth of empirical evidence to guide our understanding about how oysters adapt to salinity extremities under *in situ* conditions. To address this gap, *in situ* transcriptomes were analyzed to provide exciting glimpses into the active transcripts of samples collected in the field. Based on the PCA analysis, the total transcriptomic expression profile (P2–P5) were overlapped, which suggests that salinity levels of 8.8–22.4 ppt represent tolerable salinity limits for normal physiological activities. Beyond these limits, distinct gene expression profiles, as represented by oysters collected from P1 to P6, were found to explicitly diverge from the normal expression patterns seen for the locations of P2–P5. This transcriptomic divergence strongly suggests that adaptive gene expression networks are at work in the oysters’ response to extreme conditions of salinity. Collectively, a total of 25.4% DEGs were identified in the Hong Kong oyster under extreme conditions of hypo- and hyper-salinity across naturally formed salinity gradients, which greatly exceeds the DGEs number previously reported for stimulation conditions used in the laboratory(Meng et al., 2013; Yan et al., 2017). Implicitly, this also means that the oysters have a much greater capacity and functional plasticity for salinity adaptation in the field environments than can be envisioned for laboratory manipulations. It is difficult to rule out the possibility that certain environmental factors may interfere the final results for *in situ* analysis when compared to tightly controlled modeling experiments in the laboratory. However, validity of the current *in situ* sampling methodology is in part justified on the ground that the sampling locations all lie within a narrow span of latitude with similar climate features (e.g., temperature); no obvious disease was found within this region, suggesting that salinity likely stands out as the most important variable for this studies. It then logically follows that the extreme conditions of hypo-salinity and hyper-salinity lead to distinct expression profiles. The crucial genes and pathways involved in signal transduction, actin cytoskeleton dynamics and adhesion, metabolism and immune defense are discussed as follows.

### Signal Transduction

Strikingly, FoxO signaling pathway and oxytocin signaling pathway were observed to be mostly up-regulated by hypo-salinity. Indeed, both of these signaling pathways are conserved regulators for insulin signaling and glucose metabolism (Webb and Brunet, 2014; Elabd and Sabry, 2015; Watanabe et al., 2016), being consistent with that hypo-salinity promotes growth of oyster shell weight and size (our unpublished data). Moreover, FoxO transcription factors play a pivotal role in promoting the expression of genes involved in antioxidant defense (Klotz et al., 2015). Here, two target genes, namely, catalase and GADD45, were also increased in oysters subjected to hypo-salinity conditions. Previous evidence showed that catalase can catalyze the decomposition of hydrogen peroxide, a detrimental ROS (reactive oxygen species) when produced in excess, and protect host cells against the oxidative injury in the Hong Kong oyster (Zhang et al., 2011). GADD45 is another an oxidative stress response protein responsible for repairing oxidant induced DNA damage (Li et al., 2017). Thus, it seems reasonable to speculate that activation of FoxO signaling and its target genes may contribute to resolution of oxidative stress under hypo-salinity. It is interesting to note that extreme hypertonic stress reportedly activates FoxO signaling to result in lifespan extension (Lamitina and Strange, 2005; Anderson et al., 2016) in *Crassostrea elegans*, suggesting distinct adaptive strategies during the evolution. In contrast, hyper-salinity conditions led to inhibition of PI3K-Akt signaling pathway in the Hong Kong oyster, as evidenced by a suppression of phosphoinositide 3-kinase (PI3K), receptor tyrosine kinases (RTKs) and focal adhesion kinase (FAK). Taken into account that all of these signaling pathways are crucial for cell proliferation/growth with conserved functions in species from *Drosophila* to mammals, down-regulation of the pathways may partially account for hyper-salinity dependent growth suppression in the oysters.

### Metabolism

Many euryhaline marine invertebrates have been reported to regulate their extracellular and intracellular osmolality in response to salinity fluctuations (Liu and Hellebust, 1976; Boone and Claybrook, 1977; Pierce, 1982; Eierman and Hare, 2014). Although oysters lack the ability for adjustment of extracellular fluids, they have a compensatory machinery for transporting osmotically active solutes to maintain osmotic hemostasis (Meng et al., 2013). Among them, free amino acids (FAAs) were shown to account for the majority of the total osmotically active solutes. In this light, metabolism of amino acid is thus anticipated to be critical for osmoregulation in this marine organism. Here, the metabolism pathways responsible for arginine and proline metabolism are regulated under hyper-salinity, suggesting functional significance of the amino acids in osmoregulation. Apart from amino acids metabolism, genes responsible for riboflavin metabolism were also observed to be increased as counter-measures against hyper-salinity. While there is a lack of evidence in animals, previous studies have shown that riboflavin can enhance organismal resistance to salinity stress through augmenting organic solutes and ion uptake in plants (Azooz, 2009). So, it is tempting to speculate whether analogous mechanisms exist in oysters to enhance osmoregulation.

### Immune Defense

Other than their prototypical roles in seawater filtration, gills are also a key contributor to host innate immunity in oysters (Dautremepuits et al., 2009; Zhang et al., 2015). We observed that immune response was elicited when Hong Kong oyster encountered hypo-salinity but not hyper-salinity. Similarly, such situations have also been reported in other oyster species, such as the Sydney rock oyster and Pacific oyster (Meng et al., 2013; Yan et al., 2017), suggesting that immune response can be elicited or sustained in oysters in hypo-salinity environments (Butt et al., 2006; Gagnaire et al., 2006). Chemokine signaling pathway is one of many crucial biological responses implicated therein. Importantly, chemokines promote changes in cellular morphology, or more specifically cell polarization, to thwart pathogenic infection, typically through the activation of JAK/STAT signaling (Mellado et al., 2001). In our study, the homologs of JAK/STAT were observed to be upregulated under hypo-salinity conditions in the Hong Kong oyster, suggesting that hypo-salinity environments could activate chemokine signaling pathway. Two factors may potentially account for this process. Firstly, coastal microbial abundance and density generally surpass that in the high-salinity ocean waters (Hassard et al., 2016), which may readily trigger off immune response and activate immune signaling pathways. Secondly, hypo-salinity may aggravate the stress from cell polarization in the gills, and *vice versa*, which enhances chemokine activation. However, both of these hypotheses need further validation. Additionally, other important immune pathways, pathogenic infection and platelet activation, were observed to be adaptively regulated under hypo-salinity conditions. Platelet activation are crucial to host inflammatory and immunomodulatory activities in vertebrates (Assinger, 2014). Despite limited knowledge established on its process of platelet activation, most homolog genes are present in oysters. Of note, adenylyl cyclase, a central signaling regulator known for catalyzing the conversion of ATP to cAMP (3’,5’-cyclic AMP) and functioning as a key second massager in multiple physiological processes, was found to increase in mRNA expression under hypo-salinity. It has been recognized that cAMP signaling strongly influences host immune defense through regulating inflammatory response, phagocytosis and antimicrobial activities (Serezani et al., 2008). Additionally, cAMP improves host salinity tolerance by regulating NaCl and water absorption in aquatic animals, corroborating a dual role of cAMP in oyster hypo-salinity response (Tresguerres et al., 2014).

### Other Osmoregulation Associated Genes

In additional, salinity correlation analysis reveals several marker genes whose expression seems tightly coupled with salinity fluctuations. For instance, hypoxia-inducible factors (HIFs) are master transcriptional factors for oxygen homeostasis in all animal species, including oysters (Lee et al., 2004; Helen, 2011; Piontkivska et al., 2011; Nguyen et al., 2013). Given that the solubility of oxygen declines with increasing salinity, long-term exposure to hyper-salinity favors the induction of hypoxia response (Lange et al., 1972). Consistent with this assumption, the hypoxia inducible factor 1 α inhibitor (HIF1AN) was observed to be upregulated as salinity increased, which may contribute to mitigation of HIF over-activity and maintenance of oxygen hemostasis. Moreover, another important salinity positive correlation marker gene was identified to be cholecystokinin receptor, which has been previously reported to regulate protein and fat digestion and control of energy balance (Wank, 1995). Up-regulation of cholecystokinin receptor in our findings would suggest that increased energy demand may ensue for living in a suboptimal environment above physiological salinity. In contrast, the expression of several marker genes was negatively correlated with the salinity gradient including three homologs of LDL receptor, neuronal AChR β3 and others. LDL receptors are associated with lipoproteins transfer and are functionally important for endocytosis and vesicles trafficking (Jeon and Blacklow, 2005). These proteins are also essential for innate immune defense, since lipoprotein deficiency results in increased susceptibility to bacterial infections (Peterson et al., 2008), which is consistent with increased ease of immune activation within a low salinity environment. In additional, salinity dependent down-regulation of neuronal AChRs suggests that the neurotransmitter may be directly involved in osmotic adaptation. Neuronal AChR responds to their cognate ligands neurotransmitter acetylcholine, and trigger the activation of second messenger (Albuquerque et al., 2009). Meanwhile, as a non-selective cation channel, AChRs could transport several different positively charged ions, including Na^+^ and K^+^, to keep intracellular ion homeostasis (Dani and Bertrand, 2007).

## Conclusion

Here, our work represents a pioneering study on the regulatory mechanisms of oyster adaptive response to extreme conditions of salinity *in situ*. Distinct transcriptomic expression profiles were found among oysters living in extreme conditions across naturally formed salinity gradients. Diverse pathways including signal transduction, immune response, amino acid metabolism and others were altered in response to salinity fluctuations, revealing the striking complexity and plasticity of transcriptomic expression of oysters in eurysalinity adaptation.

## Author Contributions

YZ and SX conceived and designed the experiments. SX, JL, YL, YhZ, HM, and RM performed the experiments. SX, N-KW, YZ, and ZY analyzed the data. SX, N-KW, YZ, and YZ wrote the paper. All authors reviewed the manuscript.

## Conflict of Interest Statement

The authors declare that the research was conducted in the absence of any commercial or financial relationships that could be construed as a potential conflict of interest.

## Supplementary Material

FIGURE S1 Boxplot presents the expression profile of all samples.

FIGURE S2 MA-plot shows the DGEs under hypo-salinity.

FIGURE S3 MA-plot shows the DGEs under hyper-salinity.

FIGURE S4 The correlation of gene expression between RNA-seq and Real-Time qPCR.

TABLE S1 The transcriptome and mapping reads statistics.

TABLE S2 The gene list and functional annotation of DGEs under hypo-salinity.

TABLE S3 The gene list and functional annotation of DGEs under hyper-salinity.

TABLE S4 The list of primer used in the validation.
