# Supplementary figures and images for: Analysis of in situ Transcriptomes Reveals Divergent Adaptive Response to Hyper- and Hypo-Salinity in the Hong Kong Oyster, Crassostrea hongkongensis
Source: Front Physiol. 2018 Oct 26;9:1491. doi: 10.3389/fphys.2018.01491 (PMC6212563; doi:10.3389/fphys.2018.01491)

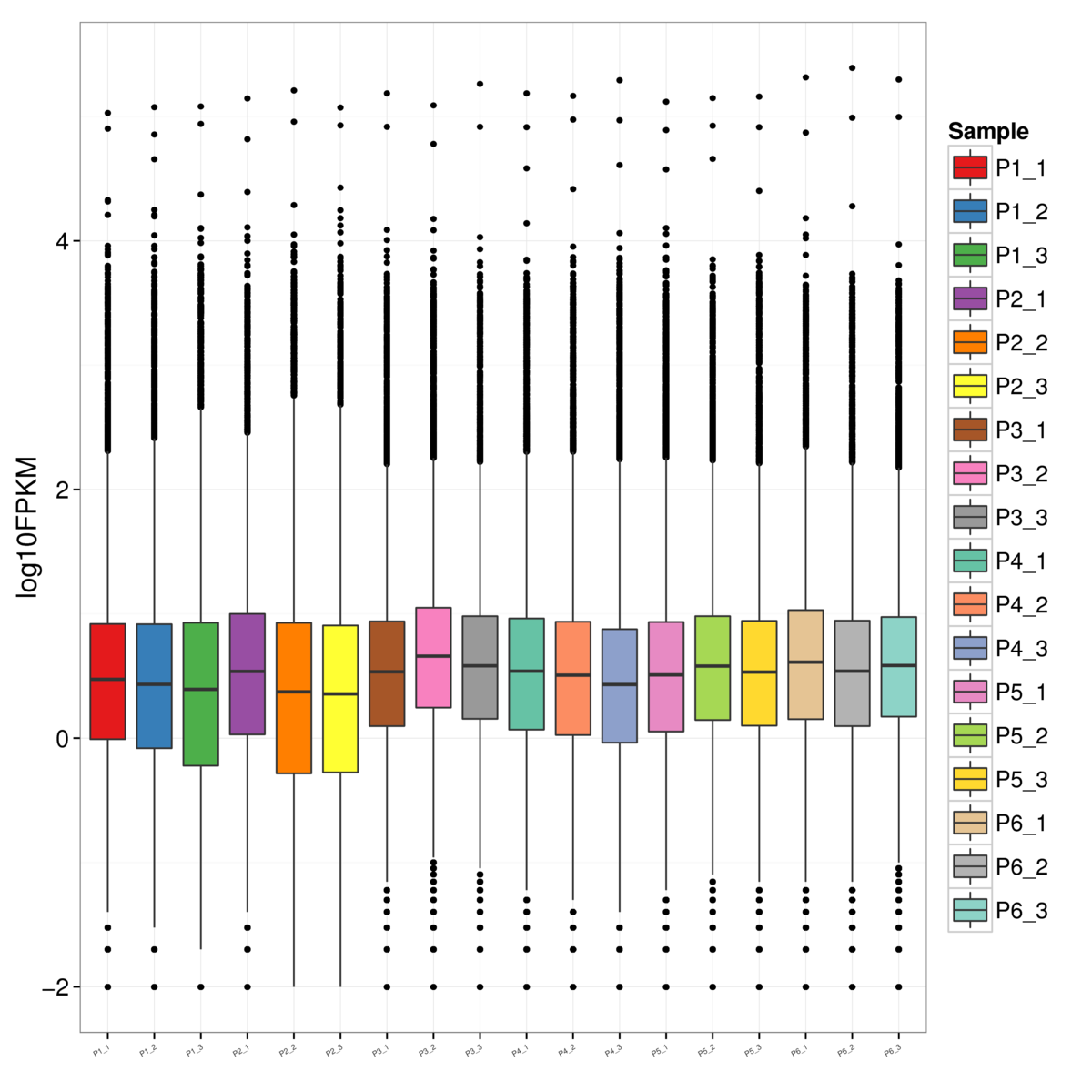

Supplement: FIGURE S1 — Boxplot presents the expression profile of all samples. [file Image_1.PNG]

# MA plot of P3-VS-P1.DEseq2\_Method

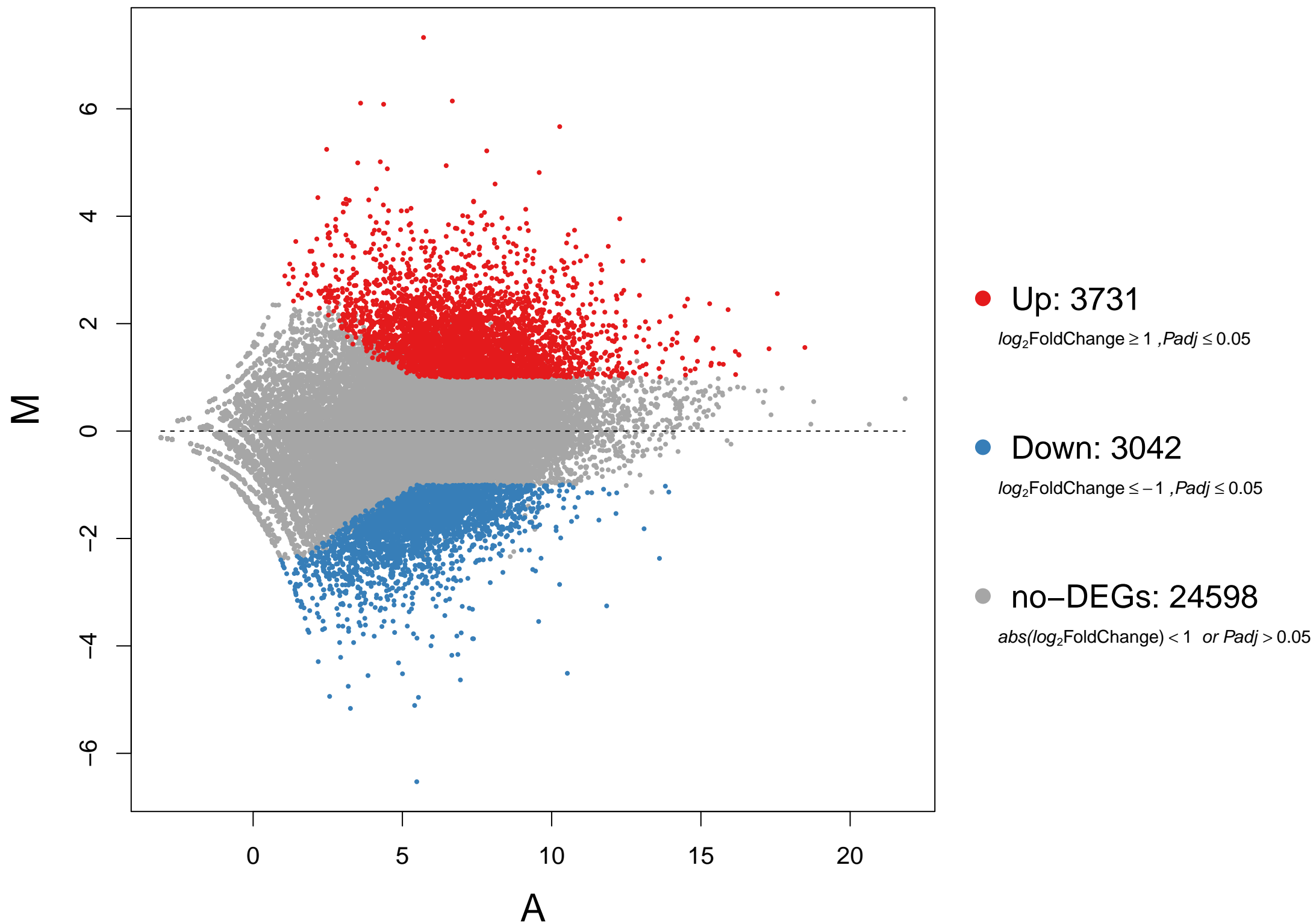

Supplement: FIGURE S2 — MA-plot shows the DGEs under hypo-salinity. [file Image_2.pdf]

# MA plot of P3-VS-P6.DEseq2\_Method

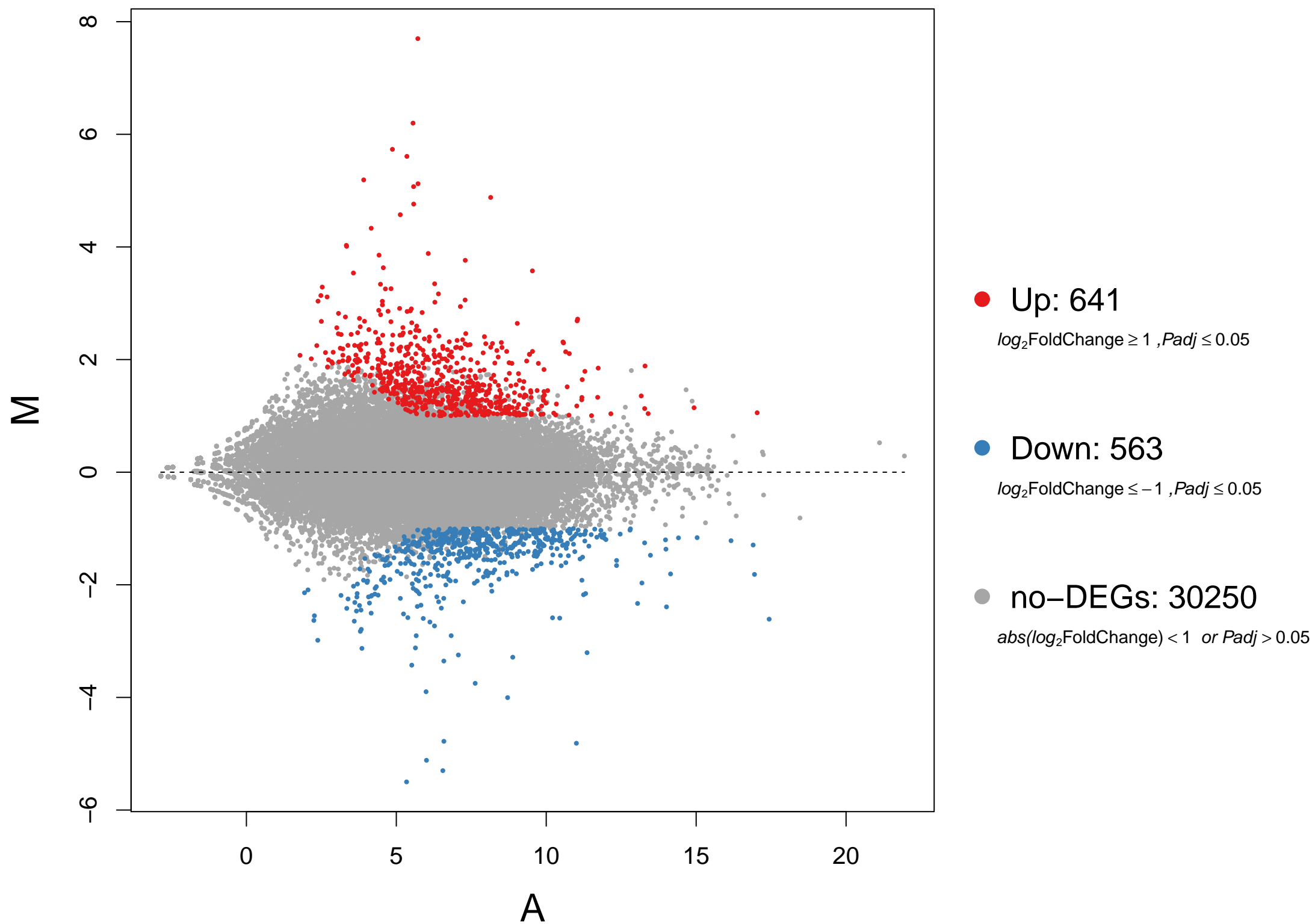

Supplement: FIGURE S3 — MA-plot shows the DGEs under hyper-salinity. [file Image_3.pdf]

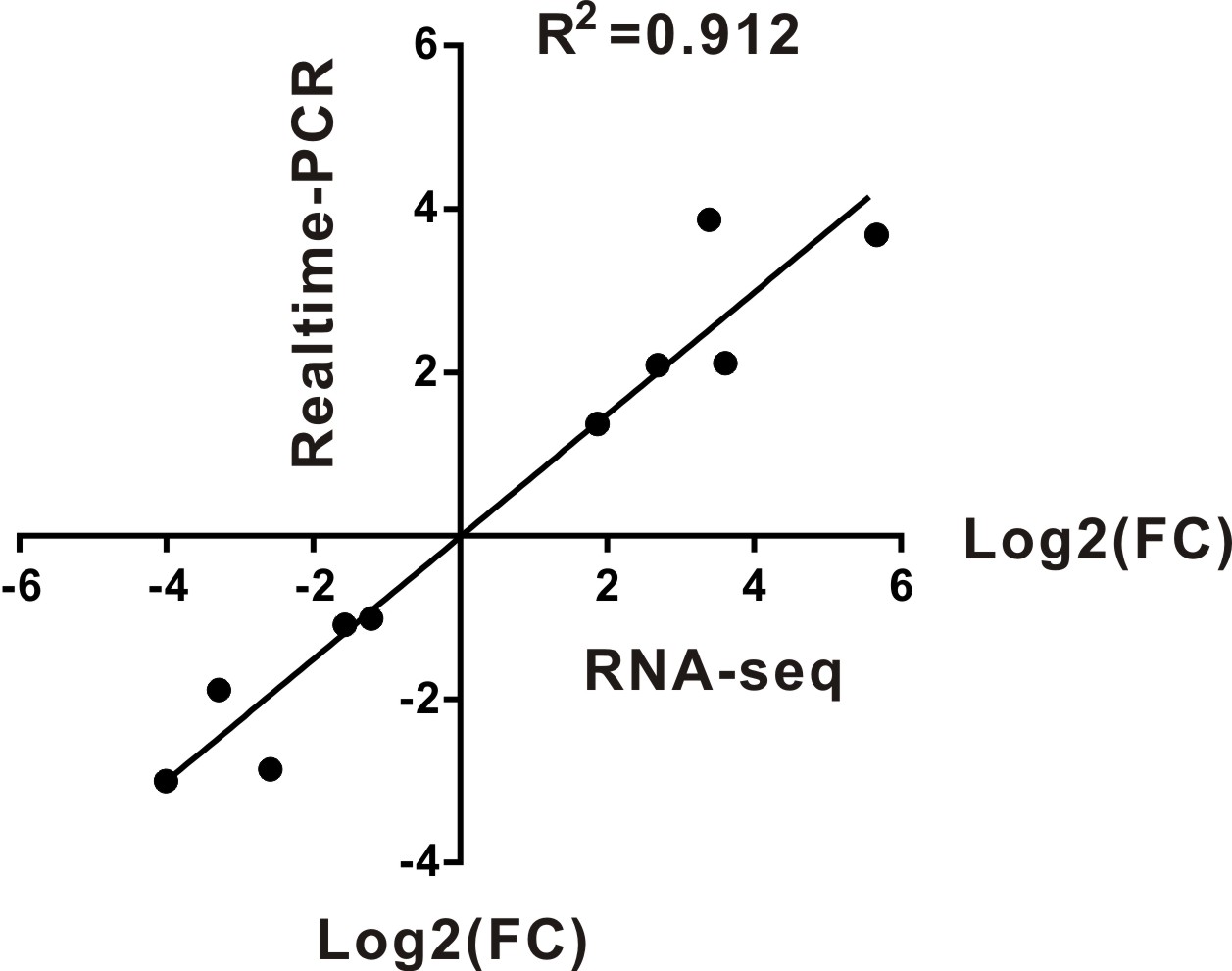

Supplement: FIGURE S4 — The correlation of gene expression between RNA-seq and Real-Time qPCR. [file Image_4.jpeg]
