# Supplementary material for: Analysis of in situ Transcriptomes Reveals Divergent Adaptive Response to Hyper- and Hypo-Salinity in the Hong Kong Oyster, Crassostrea hongkongensis
Source: Front Physiol. 2018 Oct 26;9:1491. doi: 10.3389/fphys.2018.01491 (PMC6212563; doi:10.3389/fphys.2018.01491)
Supplement: TABLE S1 — The transcriptome and mapping reads statistics. [file Table_1.DOCX]

| **Sample name** | **Total Reads** | **Total Bases** | **Total Mapped Reads (%)** | **Unique Match (%)** | **Total Unmapped Reads (%)** |
| --- | --- | --- | --- | --- | --- |
| **P1-1** | 24056323 | 1202816150 | 81.65 | 77.95 | 18.35 |
| **P1-2** | 24067597 | 1203379850 | 80.33 | 76.31 | 19.67 |
| **P1-3** | 24095353 | 1204767650 | 79.81 | 76.49 | 20.19 |
| **P2-1** | 24049067 | 1202453350 | 76.79 | 73.59 | 23.21 |
| **P2-2** | 23809498 | 1190474900 | 78.7 | 75.77 | 21.3 |
| **P2-3** | 23865845 | 1193292250 | 77.44 | 73.81 | 22.56 |
| **P3-1** | 24094905 | 1204745250 | 81.07 | 77.85 | 18.93 |
| **P3-2** | 24083135 | 1204156750 | 79.45 | 76.37 | 20.55 |
| **P3-3** | 24080724 | 1204036200 | 80.65 | 78.15 | 19.35 |
| **P4-1** | 24101605 | 1205080250 | 80.52 | 77.71 | 19.48 |
| **P4-2** | 24078663 | 1203933150 | 80.99 | 78.33 | 19.01 |
| **P4-3** | 24055560 | 1202778000 | 83.15 | 80.64 | 16.85 |
| **P5-1** | 24198896 | 1209944800 | 82.73 | 79.13 | 17.27 |
| **P5-2** | 24064127 | 1203206350 | 80.87 | 77.95 | 19.13 |
| **P5-3** | 25385871 | 1269293550 | 80.11 | 77.09 | 19.89 |
| **P6-1** | 24096214 | 1204810700 | 78.73 | 75.96 | 21.27 |
| **P6-2** | 24089448 | 1204472400 | 80.56 | 78.38 | 19.44 |
| **P6-3** | 24003997 | 1200199850 | 80.76 | 78.34 | 19.24 |
