# Supplementary material for: Analysis of in situ Transcriptomes Reveals Divergent Adaptive Response to Hyper- and Hypo-Salinity in the Hong Kong Oyster, Crassostrea hongkongensis
Source: Front Physiol. 2018 Oct 26;9:1491. doi: 10.3389/fphys.2018.01491 (PMC6212563; doi:10.3389/fphys.2018.01491)
Supplement: TABLE S4 — The list of primer used in the validation. [file Table_4.DOCX]

| Gene Accession Number | Sequence |
| --- | --- |
| EKC38551.1 | F:GTGTTTCTGCTTTTGCCTGTTCTTCG |
|  | R:ACCCTCCCCCTAACTCATTCTCGTG |
| XP_011430819.1 | F:ATAGCAAGAGCATCAAGTCGGTG |
|  | R:ACACCTACACACCAGTCGGGG |
| XP_011426293.1 | F:TTGCTTCAAAGTCTGATTCGC |
|  | R:TCTTCATTGTCCTCTACACCGC |
| EKC28954.1 | F:GCAGTAGAGACCGTAGCAATGAG |
|  | R:CAATAACAGGGATAAGTGATGAACC |
| XP_011414031.1 | F:TTTGTTGGTTCTGTTGTTCG |
|  | R:CAGAATCACTGCGTCGTAAC |
| XP_011412702.1 | F:AACTACTACGACGCCCTTCA |
|  | R:TCACTCGGAACGACTGTAAT |
| EKC33849.1 | F:AAGGCTCGTGACCGCAATGA |
|  | R:AAATCGGGGGTGGGCAACTT |
| XP_011430293.1 | F:TTGTCATACCATCATTCGCA |
|  | R:CTCCAGGGTCATTTGAAGAA |
| XP_011414812.1 | F:AGTGAAAGTCAACTGTTATGCG |
|  | R:GAAGATACTGAGGGGTAAGGGT |
| XP_011450497.1 | F:AACGAGTTCAAGCAGCACCCC |
|  | R:CTTGCCTTTGAGTGGTTTTGTCC |
| Reference Gene (GADPH) | F:GGATTGGCGTGGTGGTAGAG |
|  | R:GTATGATGCCCCTTTGTTGAGTC |
